# Ghanaian nurses' and midwives' perspectives on technology adoption in nursing and midwifery education

**DOI:** 10.1002/nop2.1342

**Published:** 2022-08-30

**Authors:** Charles Ampong Adjei, Samuel Adjorlolo, Josephine Kyei, Lillian Akorfa Ohene, Gladys Dzansi, Angela Kwartemaa Acheampong, Isabella Naana Akyaa Asante, Philomena Woolley, Felix Nyante, Lydia Aziato

**Affiliations:** ^1^ School of Nursing and Midwifery University of Ghana Legon Ghana; ^2^ School of Nursing and Midwifery Wisconsin International University College‐Ghana Accra Ghana; ^3^ Isabella HealthCare Services Accra Ghana; ^4^ Nursing and Midwifery Council of Ghana Accra Ghana

**Keywords:** computer‐based test, Ghana, innovation, midwives, nurses, online distance education, technology

## Abstract

**Aim:**

The purpose of this study was to explore the perceived benefits and challenges of online distance education and computer‐based testing (CBT) among registered nurses and midwives in a sample of government‐owned health facilities and health training institutions in Ghana.

**Design:**

Exploratory descriptive qualitative design.

**Methods:**

Individual semi‐structured interviews were conducted with 45 participants (i.e. 25 nurses and 20 midwives). The data were manually processed and analysed using Braun and Clarke’s thematic analysis approach (November 2019‐February 2020).

**Results:**

Nurses and midwives prefer online distance education for three reasons: convenience, cost‐effectiveness and learning centre proximity to the workplace. The course schedule’s flexibility allowed participants to work and study simultaneously. Others acknowledged online distance education as a viable option for overcoming the challenges of obtaining study leave. However, the lack of recognition of certificates by some employers, poor Internet connectivity and perceived excessive course load were noted as deterrents. Regarding the CBT, many of the participants said that it was useful. Among the advantages of CBT are: (1) a decrease in examination malpractices, (2) a decrease in examination costs and (3) a rise in students’ interest in information, communication and technology (ICT). This finding emphasizes the necessity of integrating ICT into nursing and midwifery education and examinations, as well as maximizing its benefits.

## 1 INTRODUCTION

In recent years, nursing and midwifery education has seen tremendous technological transformations (Jowsey et al., 2020; Smart et al., 2020; Tagoe & Cole, 2020). Computer‐based learning is gaining popularity in several countries (Jowsey et al., 2020; Letseka et al., 2018), especially Sub‐Saharan Africa (Andoh et al., 2020; Asunka, 2014; Kotoua et al., 2015; Robinson‐Bassey & Edet, 2015; Tagoe & Cole, 2020). The relevance of technological innovation in nursing and midwifery has been even more apparent since the emergence of the Covid‐19 epidemic, which has promoted the adoption of teaching and learning approaches that limit face‐to‐face contact (Jowsey et al., 2020). Furthermore, the impact of the Covid‐19 pandemic on education adds to the discussion that changing societal needs new approaches to teaching, learning and assessment. Online distance education and computer‐based tests (CBT) are the two most common technology innovations utilized in nursing and midwifery education (Christmals & Gross, 2019; Jowsey et al., 2020; Tagoe & Cole, 2020).

Online distance education refers to “teaching and learning situations in which the instructor and the learners are geographically separated and therefore rely on electronic devices and/or print materials for instructional delivery” (Knebel, 2001). The two most frequent modalities of Internet‐based distance education are synchronous (face‐to‐face) and asynchronous (text‐based) (Clark, 1998; Regmi & Jones, 2020). In synchronous education, recordings of lectures in the classroom are combined with additional resources, such as Power Point slides, and interactive platforms, such as chatlines, which allow for real‐time interactions (Clark, 1998). Synchronous online education requires that both the learners and instructors or facilitators are present concurrently. In contrast, asynchronous education does not come with a scheduled online material, quizzes and examination but does include an interactive component in the form of unscheduled written messages, audio and video posts to discussion boards, forums and blogs (Clark, 1998).

There is a preponderance of research demonstrating the benefits of online distance education (Jowsey et al., 2020; Regmi & Jones, 2020; Xing et al., 2020; Yidong & Youqiang,  2020). A systematic review, for example indicated that online distance education is more flexible, accessible and allows for academic dialogue between students and facilitators (Regmi & Jones, 2020). It also offers a convenient learning environment, rich experiences, work/life balance and easy access to learning materials for students (Jowsey et al., 2020; Jowsey et al., 2020; Yidong & Youqiang, 2020). Some scholars argue that online distance education is a viable alternative to conventional education (Letseka et al., 2018). Many African higher learning institutions have recently begun to complement traditional education with online distance education (Andoh et al., 2020; Asunka, 2014; Kotoua et al., 2015; Letseka et al., 2018; Tagoe & Cole, 2020).

Online distance education has its unique challenges, including high cost of data bundles, poor students' motivation and lack of computer skills that affect either the students (Jowsey et al., 2020; Regmi & Jones, 2020; Tagoe & Cole, 2020) or the facilitators (Sinacori, 2020). For example, flexibility and unstructured course pattern that characterize online method of education has been found to generate tension among students, leading to academic programme non‐completion (Jowsey et al., 2020). Affective‐cognitive issues such as disengagement, boredom and anxiety have also been linked to lower levels of involvement in online distance education (Wang et al., 2015). Nonetheless, Jowsey et al. (2020) argue that with high students' confidence and appropriate support, the performance of students in online distance education programmes tends to improve.

Besides online distance education, significant advancements in information, communication and technology (ICT) are increasingly replacing paper‐based evaluation methods in nursing and midwifery education with CBT (Christmals & Gross, 2019; Thurlow et al., 2010). Some researchers contend that CBT is faster and less expensive (Thurlow et al., 2010); however, others have expressed concerns regarding the logistical requirements and test anxiety associated with CBT (Al‐Saleem & Ullah, 2014; Geraili‐Afra et al., 2018). According to the findings of a quasi‐randomized control study, nurses assigned to a CBT group reported greater test anxiety scores than their counterparts in a paper‐based test group (Geraili‐Afra et al., 2018). Furthermore, in comparison to paper‐based tests, the design and implementation of CBT require more logistics and expertise for the development of item banks, methods for test delivery, procedures for test administration and test security (Al‐Saleem & Ullah, 2014).

Until recently, conventional education was the only method for nurses and midwives in Ghana to be trained. Many private and public universities provide online distance education for nurses and midwives who have completed their basic education in their respective disciplines (i.e. diploma in nursing and midwifery) and intend to pursue a higher degree in nursing and/or midwifery (Tagoe & Cole, 2020). To enhance teaching and learning, various management software such as Sakai, Google Classrooms, Zoom and Microsoft Teams are employed. In addition, Ghana’s Nursing and Midwifery Council (NMC) has recently begun online examination for certification as a nurse or midwife (Christmals & Gross, 2019). However, little is known about the challenges associated with these new learning, teaching and assessment methods, which are gradually increasing in Ghana. Although previous studies have highlighted some challenges associated with online education, the gross differences in the adoption and utilization of technology across countries necessitate country‐specific analyses to inform context‐relevant interventions. To better understand these challenges in the Ghanaian context, we designed this study to explore the perspectives of nurses and midwives who have been involved in online distance education and CBT in government‐owned health facilities and health training institutions in Ghana. Understanding these challenges would help to improve the effectiveness of these technological innovations adopted by higher institutions of learning and the NMC, as well as contribute to the cross‐cultural literature on the application of online education and CBT to nursing and midwifery education.

## 2 METHODS

### 2.1 Study design and setting

Given the dearth of evidence in the study context, the study used an exploratory descriptive qualitative design (Creswell, 2014) to gain a broader understanding of the phenomenon. Participants were drawn from three government‐owned health facilities and two health training institutions.

### 2.2 Participants and recruitment

The study purposely recruited 15 registered general nurses (RGN), 11 registered midwives (RM), 10 registered mental nurses (RMN) and 9 health educators. Participants were included if they had taken a CBT or participated in an online distance education programme in nursing or midwifery. The participants were invited to participate in the study following a visit to their respective facilities by the research team. The purpose and voluntary nature of the study were discussed with the potential participants. On average, seven participants were recruited from each of the six regions. Out of the 45 participants, 36 were selected from health facilities and 9 from tertiary nursing and midwifery training institutions. The prolonged engagement as well as the recruitment of more participants assisted with an in‐depth understanding of the phenomena. None of the participants contacted who satisfied the inclusion criteria refused to take part in the study.

### 2.3 Data collection procedure

Data were collected between November 2019–February 2020 following ethical approval and permission from the selected health facilities and the educational institutions. To recruit the nurses and midwives from the hospitals, the research team visited some departments including the medical, surgical, out‐patient, theatre, mental health, public health, accident and emergency departments. First, the nurse and midwife managers were informed about the purpose of the study. The unit/department managers assisted the research team to recruit eligible participants. In the health training institutions, permission was sought from the heads of the institutions. Afterwards, potential participants were contacted and briefed about the study purpose and measures instituted to ensure the confidentiality of their information. Potential participants who consented to take part in the study were engaged in a face‐to‐face individual interview at the offices of the nurses/midwives and the health educators, respectively. Overall, two interviewers with a master’s level qualification and a PhD and a qualitative expertise were involved in the data collection. All the interviews were audio‐recorded following permission from the participants. Also, field notes were taken. The interviews lasted between 45 min–1 hr.

### 2.4 Research instrument

A semi‐structured interview guide was developed based on the literature review and study objectives. The following topics were largely explored throughout the interview: (1) nurses' and midwives' perceptions of online distance education and CBT and (2) challenges that nurses and midwives face with online distance education and CBT. These questions were probed further in order to have a thorough understanding of the phenomenon.

### 2.5 Data processing and analyses

The data were manually processed. The procedure for thematic analysis described by Braun and Clarke (2006) was followed. First, all of the audio recordings were verbatim transcribed. Second, two members of the study team coded the transcripts, followed by the identification and definition of themes that emerged from the compiled list of codes. Third, the research team had a series of talks until consensus was reached on the themes. Fourth, preliminary findings were reviewed with two representatives of nurses/midwives and health educators to ensure that they were congruent with their views.

### 2.6 Ethics and rigour

The Ghana Health Service Ethics Review Committee granted ethics approval (Protocol No. GHS‐ERC/011/05/19). Each participant signed a written informed consent form. Furthermore, it was highlighted that they had the right to withdraw from the study with no consequences. Field notes were obtained to ensure rigour and confirmability. A clear description of the research process ensured dependability. A detailed description of the study settings ensured transferability. To ensure that the information gathered from the participants was credible, a probing technique was used (Polit & Beck, 2014).

## 3 RESULTS

### 3.1 Participants' demographic characteristics

Participants included RGN, RM, registered mental health nurses and health educators from six regions in Ghana. All of the educators worked as tutors at nursing or midwifery training schools. Their ages range from 27–59 years. The participants' years of experience in their respective fields range from 2–33. Thirty‐one of the 45 participants recruited were females, with the remaining 14 being males. The study primarily identified two major technology innovations in nursing and midwifery education. One of these innovations is online distance education, and the other is computer‐based testing (CBT). All of the participants worked full‐time and were not on leave to study. Furthermore, all of them were enrolled in an undergraduate programme that included virtual and face‐to‐face sessions (weekend tutorials).

### 3.2 Online distance education

The first technology adopted in nursing and midwifery education that most participants mentioned was online distance education. The following two major themes emerged: (1) enablers (such as convenience, cost‐effectiveness and proximity of learning centres to workplaces) and (2) challenges associated with online distance education (such as certificate recognition, Internet accessibility and perceived high course load). A summary of the themes and sub‐themes is presented in Table 1.

**TABLE 1:** Themes and sub‐themes – Online distance education

Theme	Sub‐theme
Enabling factors of online distance education	Convenience Cost‐effectiveness Learning centres' proximity to the workplaces
Online distance education challenges	Certificate recognition Internet accessibility Perceived excessive course load

#### 3.2.1 Enabling factors of online distance education

Three major factors influencing participants' choice of online distance education were convenience, cost‐effectiveness and the closeness of the learning centre to the nurses' and midwives' places of work.

##### Convenience

Almost all participants considered online distance education as more convenient than traditional education. The course schedule’s flexibility enabled participants to maintain their jobs while also pursuing higher education. The participants from northern Ghana were especially pleased that the online education pathway had removed the geographical barrier that had hitherto impacted their professional growth. The long journey to school in southern Ghana was no longer a problem:

I am attending the e‐learning distance programme in one public university in Ghana. I didn’t have to vacate post or travel from the north to Accra to attend lectures. I follow the course using my phone and computer online. RMN, Northern Region

You know because we are working, it is very difficult for you to leave your work and travel down south to attend school. The e‐learning or distance programs have come at the right time. Now nurses can be at work and at the same time be attending school. RGN, Upper East Region

Some participants indicated that online distance education is a solution to the difficulties associated with acquisition of study leave. According to them, the demand to serve a facility for many years before permission is granted to pursue an academic programme has been reduced by online distance education:

Going to do my bachelor after my diploma nursing programme was not an issue at all. I didn’t apply for study leave, never sought permission from anyone but here I am with my degree from the University of Ghana. RM, Ashanti Region

Almost all participants emphasized the convenience of getting the course materials in advance. They stated that having access to the resources enabled them to study at their own pace and participate in discussions with their peers:

You get to have all the PowerPoint, lecture notes, and videos right at the start of the semester. This allowed some of us to plan our study time and also seek the help of others if some concept is unclear to us. RGN, Greater Accra Region

Some participants assert that the face‐to‐face tutorial component of the online distance programme was also convenient. According to them, it was easy to obtain permission over the weekend to attend the tutorial sessions and work extra hours during the weekdays to compensate for the time spent in school:

My unit head was aware of my programme and she gladly gave me a weekend off at all time to attend the tutorials at the learning centre. What I did was to close at 4 pm during the weekday instead of the usual closing time of 2 pm. RM, Greater Accra Region

##### Cost‐effectiveness

The universities' flexible fee payment policies were cited as a significant factor in the participants' decision to pursue online distance education. According to them, some universities give a 50% discount on the first semester’s tuition, which is not available in the regular mode:

As a salaried worker with additional responsibilities, I felt that the offer by the university to accept 50% of the fees at the beginning of the school session was good. It allowed some of us to save in bits to settle the fees. I am sure all my colleagues were very happy about this as well. RGN, Northern Region

Some participants indicated that students who pursue nursing programmes online do not incur the costs associated with regular programmes, such as the hostel fee, internal and external transportation costs, and feeding. Some participants also claimed that the cost of printing and photocopying lecture notes was no longer a burden because the course instructors shared electronic versions of the materials:

No money is spent on transport, hostel, and even feeding because I am still leaving my normal life here. Staying in my accommodation and only walk some few distances to the learning centre when I feel like attending a tutorial session. RM, Northern Region

No need to pay for printing and photocopying of lecture notes because the soft copies are made available on time. You can also sit here and access the school library for information. RGN, Volta Region

##### The learning centre’s proximity to the workplace

Several participants stated that the learning centre where tests are held at the end of each semester was closer to their workplace or residence. This contributed to their decision to enrol in the programme:

I work at the Accra mental hospital and the distance from my workplace to the learning centre is just some few minutes by transport. I sometimes go write my exam and still make it to work. The closeness informed my choice of the programme. RM, Greater Accra Region

Several participants stated that the learning centre’s proximity to their place of employment made it easy for them to go in and write the exam without obtaining permission from their superiors:

I do not remember the last time I sought permission from my in‐charge to go write the exam. I only ride 3 minutes to the centre and in a few hours, I am back to work. That is how helpful the closeness is to me. RGN, Northern Region

Well, the centre is closer to most of my colleagues. I am yet to hear a complaint from any of my colleagues about the distance to the centre where exams are written. Besides, this happens during the weekends where there is less car traffic in town. RM, Greater Accra, Region

#### 3.2.2 Online distance education challenges

The participants identified three major issues related with online distance education. These include the fact that some employers do not recognize certificates, poor Internet access and a perceived excessive course load.

##### Certificate recognition

One of the primary concerns highlighted by the majority of participants was the lack of recognition by certain institutions in Ghana of certificates received through the online programme. The majority of participants described instances in which certifications from online programmes were not recognized by certain hospitals for upgrade and promotion:

Some of us completed online distance nursing courses using our off days and leaves. Now we have our certificates, but the hospital authorities refused to upgrade us when we submitted to them. RGN, Upper East Region

Others argued that schools should connect with relevant stakeholders, such as employers, to help them understand the curriculum’s content and thus enhance their confidence in the programme. The participants believe that this will result in the acceptance of certifications earned by personnel who complete online distance education courses:

It’s so strange to be told that a certificate issued by a credible institution cannot be accepted for an upgrade simply because I used my own time to go to school. This is ridiculous. Is it not rather good that I remained at the post and still went to school? Those who matter in this need to be talked to seriously. RM, Greater Accra Region

However, some of the participants indicated that complementing the online teaching method with the traditional classroom approach may yield a better outcome:

I feel that the institutions should structure the programme such that we do about 70% online and go do 30% in a module for a period of say 1 month every semester. Maybe the managers of the hospital will be comfortable with that. RGN, Western Region

##### Internet accessibility

All participants stated that having a poor Internet connection is a major challenge. The participants described instances in which online mid‐semester examinations were disrupted due to a bad Internet connection. According to reports, such incidents frequently have an adverse effect on students' performance in examinations:

We write the mid‐semester examination using the Sakai online platform. In many instances, I had to request for re‐assessment because the internet went down in the middle of the examination. This is one of the problems I will say about the online programme. RM, Ashanti Region

Sometimes in the course of writing mid‐semester exams, the system time you out on it own. It’s always frustrating. RGN, Volta Region

Additionally, participants experienced delays in responding to their requests for assistance with Internet problems during the assessment. According to them, the method for filing complaints is insufficiently responsive. As a result, individuals were occasionally required to travel to the university’s main campus to have their issues addressed:

When you have issues regarding time out of examination, it’s always difficult to get it solved. Although we have a complaint form, I do not see its effectiveness in most cases. RMN, Western Region

Alongside institutional Internet problems, some participants reported having difficulty accessing the Internet at home for self‐study. That the location of their homes did not provide sufficient Internet strength for downloading course materials and seeing instructor‐shared videos:

I struggle to download course materials and videos shared on Sakai because for unknown reasons none of the telecommunications internets offers us a good signal in my area. I have to frequently visit the internet café to get some of these materials downloaded and to check my emails as well. RGN, Northern Region

##### Perceived high course load

Some participants stated that the nursing programme’s courses for one semester are comparatively greater than other programmes such as psychology in the same online distance education. The participants claimed that the minimum number of courses every semester was nine, which they felt was excessive:

Our programme is too rigid. Too many courses to do every semester which also put a lot of pressure on us. Those doing courses such as psychology and sociology do not have such a huge course in their syllabus. RGN, Western Region

I remember the least course we did for one semester was 9. Can you imagine doing 9 courses in a semester, working alongside, and also performing family functions? It is not easy at all. RM, Ashanti Region

Others stated that the heavy course load resulted in a high level of demand during the examination. That they were required to write at least three papers per day with only a two‐hour break in between:

Because we have so many courses, we do write so many exams as well. What is disturbing is the three papers that we write in a day. It’s is more than a marathon. We are only given 2 hours break after each paper to rest for the other ones. You end up so tired. RGN, Western Region

### 3.3 Computer‐based test

In addition to online distance education, CBT was the second technological innovation in nursing and midwifery in Ghana. Two main themes were identified. These include the perceived benefits of CBT and challenges associated with CBT (Table 2).

**TABLE 2:** Themes and sub‐themes – Computer‐based test

Theme	Sub‐theme
Perceived benefits of CBT	Minimized examination malpractice Reduced cost of examination Increased desirability of ICT knowledge by students
Challenges associated with CBT	Poor network connectivity Risk of system hacking

#### 3.3.1 Perceived benefits of CBT

Majority of the participants indicated their endorsement for CBT compared to the paper‐based test. According to the participants, CBT has the potential of minimizing examination malpractices, reducing cost in conducting an examination and increasing desirability of ICT knowledge by students.

##### Minimized examination malpractice

The majority of participants believed that the implementation of computer‐based tests would reduce examination malpractices. Unlike paper‐based tests, which are regarded to be related to “cheating” during an examination, the majority of participants believe that CBT will reduce the instances of examination malpractice that have been associated with paper‐based tests in the past. One participant said:

The malpractices of cheating, stretching of neck to see someone’s work during an examination will be a thing of the past. With online exams, you can’t see someone’s answer to copy. RM, Ashanti Region

Undeserved favouritism by some examiners was also highlighted as a serious examination misconduct. Some examiners have been observed manipulating student examination marks out of sympathy, even when the student performance is abysmal. This is shown by a comment from a nurse educator who admitted to participating in such acts in the past. However, she went on to say that with the implementation of CBT, such misbehaviour will no longer be tolerated. She recalled:

Those days you come and sit down and add up the student scores and out of kindness you help him or her…, but this one (computer‐based test) when you put the score and it’s gone there’s nothing like a human face. Nurse educator, Northern Region

Another participant also said:

The marking of essay questions has some element of subjectivity. The examiner has to try and understand the meaning of what the student has written and judged it using the marking scheme, but as for MCQs (multiple choice questions) if the answer is A it is A. No one can change it, if you are correct you are correct, if you are wrong you are wrong. And after that, no one can alter it. Nurse educator, Greater Accra Region

Some participants felt that the Nursing and Midwifery Council of Ghana (NMC) should not be the primary organization in charge of conducting CBT for nurses and midwives if the methodology is to be fully utilized. What can be learned from countries such as the United States and the United Kingdom, where reputable IT companies such as Pearson Vue administer licensure exams on behalf of the NMC:

When you go to places like America where online examination are taken, it is not the American nursing administration that organizes those exams. When you go to the UK where the nursing exam is online it is not the nursing and midwifery council that conduct these exams. For example, Pearson Vue is the organization that conduct these exams because it is an IT organization that has the check and balances in place that nobody can hack into their system. So why couldn’t NMC with all the monies be the first Africa institution to learn from these best practices. Nurse Educator, Northern region

##### Reduced cost of examination

Participants asserted that instituting an online examination would lower the cost of hiring invigilators for the licensure examination. Previously, the NMC of Ghana engaged the services of examiners. According to the participants, these examiners are assigned to all regions in Ghana as invigilators. Following the examination, the NMC hires additional examiners to mark the examination scripts. The expense of the exercise is believed to be enormous, given that each examiner must be transported, paid per diem and received allowances. The majority of the participants who were nurse educators stated that adopting CBT as the new evaluation method would save the council a significant amount of money due to the reduced participation of human factors:

The risk and the money involved in transporting examiners from their various locations to exam centres and to Accra to mark written exam will reduce. Nurse Educator, Ashanti region

Some of the nurse educators who were hired as examiners complained about delays in receiving their payment after the marking process was completed. According to several of the aforementioned participants, the NMC does not compensate them for their time commitment. They believe, however, that the implementation of the CBT will put an end to the recurring appeal that requests reimbursement for work done by examiners:

All the time you see petitions by the examiners requesting for payment of their allowance after supervising NMC exam. All these stories will be no more once the examination is conducted online by the council. In effect, the council will save a lot of money and probably use it for something else. Nurse Educator, Upper East Region

Another significant benefit raised by participants is the financial relief that the CBT is projected to bring to students. Specifically, almost all of the participants stated that the paper‐based test was expensive. The costs were divided into two categories: direct and indirect. The participants also stated that the CBT will considerably lower the cost of the examination as well as the expense of travelling to the regional council offices to check the results:

With the online examination, students now spend less money on transportation to check results. I am aware of an incident whereby some students who were going to check their results had an accident and died and others suffered various forms of injuries. All these have economic implications on the student and the family as a whole but with the coming into being of the online examination, all these will be a thing of the past. Nurse educator, Western Region

##### Increased desirability of ICT knowledge by students

Several participants indicated that the NMC’s implementation of the CBT will stimulate the interest of nursing and midwifery students in learning about and taking seriously ICT courses while in school. It is worth noting that ICT is included in the diploma curriculum for nursing and midwifery. The instructional period, on the other hand, is limited to a semester, with fewer hands‐on tutorials. As a result, some participants believe that any effort made to gain ICT knowledge will be increased in light of its importance in determining a student’s performance on the licensing examination:

The online examination will help those (students) who do not have an idea about ICT to take the course seriously and upgrade themselves because it’s matters in the final exams. RMN, Greater Accra Region

it is not all of us who are computer literates, even some of us were forced to learn the computer as soon as we got the hint that the examination was going to be done online. RMN, Upper East region

Some participants, however, acknowledged that although the CBT is good, as first‐time users, some difficulties were encountered by them. The limited expertise in ICT, according to the participants, made them nervous and impacted their performance one way or the other. Some are of the view that the early adoption of computer‐based assessments from the first year of school may be advantageous:

Because a lot of us are not used to computer, it was very difficult to accept the online exam. We were very anxious. I think our fears were justified because if you have never used a computer and then you are going to use it to write exams it’s won’t be easy. RMN, Northern Region

…The online examination should start the day we (student) enter the training schools so that whatever exams we write they use the internet to perfect us before the final examination. RMN, Ashanti Region

Another noteworthy report from several of the participants was about the impact of their school‐based ICT expertise on their practice. We discovered that some of the participants who worked in health institutions with electronic health systems attributed their ease of usage to the challenge CBT offered to them while in school to learn ICT:

…nowadays when you come to the ward, we use a computer, we put client’s information and everything on the computer. I feel that the demand on us to learn ICT for the final council exam have made the use of the e‐health here easier for me. So if we continue it (online examination), every nurse will be aware that we are in a digital age and that they cannot be left behind. RMN, Western Region

#### 3.3.2 Challenges of CBT

Poor network connectivity and the potential risk of system hacking were mentioned as challenges associated with CBT.

##### Poor network connectivity

The majority of participants recognized inadequate Internet access as a potential issue that could threaten the success of the online examination. Some people believed that poor Internet connectivity would have a negative impact on the examination process. Others believed that an inconsistent Internet connection could stall the examination’s progress, causing students to become anxious:

Can you imagine that during examination you experience this network problem, it will affect you mentally as a student. Health Educator, Northern Region

You won’t be ok if you are in the middle of the examination or even done answering the questions and when you are about to submit and your internet start buffering. The anxiety alone can affect your performance. RMN, Greater Accra Region

Participants from the northern region encountered an exceptional issue with low internet connectivity in regions with nursing and midwifery schools. According to the participants, accessing the internet to read articles and send emails has always been difficult, and as a result, they believe that students in these locations will be disadvantaged if examinations are conducted digitally. Two individuals shared their stories:

I was working in a very remote area where a school is located. Even opening an email, we were having some problems how much more conducting online exams, and this is one of the accredited centres for the NMC online examination. To successfully conduct an online exam, you need a very stable internet service. Health Educator, Northern Region

My problem with the online is the kind of internet access that we get because sometimes you will have the signal for 4G but you cannot download a picture within five minutes. If you have this and you want your students to write exams whereby there is a time set up for you to use and finish and then you click and the thing is rolling, you can imagine what would happen at the end. Health educator, Upper East region

Along with inadequate internet connectivity, some schools reported having fewer functional computers and accessories. It was reported that the student population does not correspond to the school’s computer quantity:

Do the training institutions have the resources: the computer, the computer labs, and the network? Health educator, Ashanti region

Apart from the internet, another key issue is getting access to computers in various institutions or centres of examination. Getting adequate computers based on the number of students is a big issue. RMN, Northern region

##### Potential risk of system hacking

Another issue raised by several of the participants was the possibility of a security flaw in the examination database. The majority of participants expressed concern about the NMC’s ability to protect examination questions from online fraudsters and hackers. A nurse educator was uncertain that NMC had the necessary cyber security to secure the examination questions over time:

Are they (NMC) not aware that in this era of IT and technological advancement someone can sit in his bedroom and hack into their systems? Are they not aware that after years of this examination the so‐called questions that they will be reshuffling becomes past question? I am telling you the solidity of the online exams are not going to stand the test of time because NMC does not have what it takes to plan online exams. Health educator, Northern Region

Others felt that stringent security measures were urgently needed to avoid internet hackers. It was requested that a contingency plan be put in place to deal with any eventuality, including a cyber‐attack:

I think that they (NMC) can put in an adequate security system to ensure that the website is not broken into for people to get access to the questions. Even if it does happen, they can have contingency measures to change the questions at any time, in such a way that, new questions can be loaded onto the page for the student to do. I believe that is far better than the pushing of papers around. Health educator, Greater Accra Region

Although almost all of the participants advocated for a strong system to secure the examination database, it was widely acknowledged that the cost of such services is prohibitively expensive. Given the high cost of guaranteeing cyber‐security, participants were concerned that the NMC would pass the fee on to students, which they saw as a disincentive:

Cyber‐security in itself is good, but it may make the online exam so expensive for the students. …Obviously, the council will ask the students to pay those cost which will be a burden. Health educator, Ashanti Region

## 4 DISCUSSION

We explored the perspectives of nurses and midwives regarding online distance education and CBT in Ghana. It was discovered that while online distance education was considered beneficial due to its convenience and cost‐effectiveness, it was negatively impacted by the absence of certificate recognition by some employers, poor internet access, and perceived heavy course load. Furthermore, CBT was recognized as necessary by participants owing to its advantages of minimizing test malpractices, reducing examination costs, and enhancing students' desire for ICT knowledge. However, challenges such as poor internet connectivity and the potential for system hacking were raised.

First, the participants' perceptions of the benefits of online distance education are expected given Ghana’s inequitable distribution of higher learning institutions offering nursing and midwifery education. For example, universities offering nursing and midwifery programmes are located in around six of Ghana’s sixteen administrative areas. As a result, prospective students are compelled to travel to one of these places in order to pursue higher degrees. Notably, the majority of nurses and midwives work in communities, districts and municipalities that are not part of the regions served by these universities. As a result, online education is the preferred method of bridging the geographical gap. Similarly, to our findings, other studies have highlighted the convenience that online distance education provides students (Andoh et al., 2020; Tagoe & Cole, 2020), including the ability to combine employment and study (Jowsey et al., 2020).

The absence of a study leave requirement as a prerequisite for enrolment in online education has also aided in the attractiveness of online education programmes. Acquiring study leave for continuous learning is a significant challenge in Ghana (Asamani et al., 2019; Kwamie et al., 2017). Almost all healthcare institutions require a minimum of 3 years of continuous service before granting study leave. In some health care facilities, study leave is considered a privilege rather than a right of an employee, even if the individual meets the required prerequisites for study leave. We add to the contention that the flexibility to participate in and study online education without the approval of an employer provides a unique opportunity for many Ghanaian nurses and midwives. Additionally, online distance education was regarded as cost‐effective by almost all the participants. This is mostly because non‐tuition‐related expenses such as hostel housing and utilities are not included in the cost of online education (Tagoe & Cole, 2020).

Besides the online distance education, our participants deemed CBT necessary due to its multiple benefits. While the outbreak of the Covid‐19 epidemic has emphasized the importance of technology advancements in nursing and midwifery education (Jowsey et al., 2020), unreliable Internet access, particularly in Africa, appears to be limiting the full value of online platforms (Tagoe & Cole, 2020). Additionally, because nursing and midwifery is a practice‐based field of study, digital assessment of students is deemed insufficient due to the accompanying restriction of doing a hands‐on activity. It is worthwhile to examine video assisted testing of clinical skills among nursing and midwifery trainees in Ghana.

Despite the aforementioned, it is worth noting that Ghana’s online distance education programme faces some notable challenges. Among them is the absence of recognition by some employers of certifications gained by nurses and midwives through online distance education. We believe that the limitation of information about the accreditation status of institutions of higher learning that provide online distance education programmes contributes significantly to this problem. Nonetheless, we believe that due diligence can be used to verify the source of an employee’s degree received through online programmes conducted locally or internationally.

The findings of this study should be interpreted within the context of several limitations. The first is the possibility of recall bias. Second, considering the qualitative approach used, caution should be exercised when generalizing the findings to all regions of Ghana.

Nurses and midwives prefer online distance education for three reasons: convenience, cost‐effectiveness and learning centre proximity to the workplace. The course schedule’s flexibility allowed participants to work and study simultaneously. Others saw online distance education as a viable option for overcoming the challenges of obtaining study leave. However, the lack of recognition of certificates by some employers, poor Internet connectivity and perceived excessive course load were noted as deterrents. Regarding the CBT, many of the participants said that it was useful. Among the advantages of CBT are: (1) a decrease in examination malpractices, (2) a decrease in examination costs and (3) a rise in students’ interest in ICT. This finding emphasizes the necessity of integrating ICT into nursing and midwifery education and exams, as well as maximizing its benefits.

## 5 CONCLUSION

This study found that many nurses and midwives prefer online distance education because it allows them to continue their education while keeping their job. Additionally, it eliminates barriers to higher education, particularly for those working in rural and hard‐to‐reach areas and provides a level playing field for their career growth. The implication is that online distance education could reduce nurses' and midwives' refusal to work in remote areas, as well as staff turnover in those places. Incorporating ICT into nursing and midwifery education and examinations, as well as the importance of taking steps to ensure an optimal outcome, is crucial in the study settings.

## AUTHOR CONTRIBUTIONS

The study was conceptualized by all the authors. Data collection was done by LA, CAA, SA, JK, LAO, GD and AKC. The manuscript was drafted by CAA and reviewed by all the authors. All authors read and approved the final manuscript.

## ETHICAL APPROVAL

Ethical clearance was obtained from the Ghana Health Service Ethics Review Committee (GHS‐ERC 011/05/19).

## CONFLICT OF INTEREST

None of the authors reported any conflict of interest.

## Data Availability

All data generated or analysed during this study are included in this article.
